# A Tug-of-War Model Explains the Saltatory Sperm Cell Movement in *Arabidopsis thaliana* Pollen Tubes by Kinesins With Calponin Homology Domain

**DOI:** 10.3389/fpls.2020.601282

**Published:** 2021-02-16

**Authors:** Saskia Schattner, Jan Schattner, Fabian Munder, Eva Höppe, Wilhelm J. Walter

**Affiliations:** ^1^Department of Biology, Institute for Plant Science and Microbiology, University of Hamburg, Hamburg, Germany; ^2^Department Medicine, Health and Medical University Potsdam, Potsdam, Germany

**Keywords:** intracellular transport, cytoskeleton, kinesin motors, pollen, sperm cells

## Abstract

Upon pollination, two sperm cells are transported inside the growing pollen tube toward the apex. One sperm cell fertilizes the egg cell to form the zygote, while the other fuses with the two polar nuclei to form the triploid endosperm. In Arabidopsis thaliana, the transport of the two sperm cells is characterized by sequential forward and backward movements with intermediate pauses. Until now, it is under debate which components of the plant cytoskeleton govern this mechanism. The sperm cells are interconnected and linked to the vegetative nucleus via a cytoplasmic projection, thus forming the male germ unit. This led to the common hypothesis that the vegetative nucleus is actively transported via myosin motors along actin cables while pulling along the sperm cells as passive cargo. In this study, however, we show that upon occasional germ unit disassembly, the sperm cells are transported independently and still follow the same bidirectional movement pattern. Moreover, we found that the net movement of sperm cells results from a combination of both longer and faster runs toward the pollen tube apex. We propose that the observed saltatory movement can be explained by the function of kinesins with calponin homology domain (KCH). This subgroup of the kinesin-14 family actively links actin filaments and microtubules. Based on KCH's specific properties derived from *in vitro* experiments, we built a tug-of-war model that could reproduce the characteristic sperm cell movement in pollen tubes.

## Introduction

A major step in the evolution of land plants was the shift from a gametophyte-dominant to a sporophyte-dominant life cycle in flowering plants which represent more than 90% of all land plants today. The male and female gametophytes of flowering plants are highly reduced and live and reproduce almost entirely within sporophytic tissues such as the anther, carpel, and ovule (Williams and Mazer, 2016). Consequently, the sperm cells have to be transported toward the ovary through the female tissue to finally fertilize the egg cell and the central cell of the megagametophyte. This challenge is met by the displacement of self-propelling sperm cells by pollen-mediated transport of the male gametes.

During maturation, the pollen undergoes two asymmetric cell divisions resulting in three cells: the vegetative cell, which encloses the two smaller generative cells—the sperm cells. As soon as the mature pollen encounters the stigma, it starts to grow a pollen tube. This tube grows through the style toward the ovary. During this process, the two sperm cells are transported inside the pollen tube toward the tube apex. As soon as the pollen tube reaches an unfertilized egg cell, it bursts open and releases the two sperm cells. Whereas, the processes of the growing pollen tube (Lord and Russell, 2002; Moscatelli and Idilli, 2009; Onelli and Moscatelli, 2013) and the fertilization process itself (Lord and Russell, 2002; Mori and Igawa, 2014; Takahashi and Igawa, 2019) are well-studied, surprisingly little is known about the transport process of the two sperm cells inside the pollen tube. In most flowering plants including the model plant *Arabidopsis thaliana*, the sperm cells travel together and are interconnected with each other as well as with the vegetative nucleus by a cytoplasmic projection (Lalanne and Twell, 2002). This complex which is composed of the two sperm cells and the vegetative nucleus is called the male germ unit (Jensen and Fisher, 1970; Russell and Cass, 1981). It appears to be quite unstable since a slight increase of temperature during *in vivo* pollen germination assays causes a disruption of the male germ unit and leads to individually transported sperm cells (Ge et al., 2011). During transport through the pollen tube, the sperm cells do not move continuously in the direction of the apex but perform saltatory back and forth movements (Hamamura et al., 2011).

Motility inside cells is most often achieved by the action of motor proteins of the myosin and kinesin superfamilies (Nebenführ and Dixit, 2018). Whilst myosins are binding to and moving along actin filaments, kinesins are associated to microtubules. Kinesins with calponin homology domain (KCHs), which are a subgroup of the microtubule minus end-directed Kinesin-14 family (Endow and Waligora, 1998), increasingly gained attention over the last years (Schneider and Persson, 2015). They are characterized by an internal kinesin-14 motor domain flanked by coiled-coil domains and an N-terminal calponin homology domain.

*In vitro* assays with the KCH1 (Walter et al., 2015) and KCH2 (Tseng et al., 2018) from *Oryza sativa* revealed that KCHs are microtubule minus end-directed motors that bind to actin filaments via their calponin homology domain and transport them along microtubules with two distinct velocities. Walter et al. (2015) proposed that the orientation-dependent transport velocities emerge from the KCHs' low torsional compliance combined with an inherently oriented binding to the actin filament.

Phylogenetic analysis of the *Arabidopsis* Kinesin-14 motor proteins revealed a subgroup of seven KCHs (Reddy and Day, 2001). Four of the seven KCHs (At1g63640, At5g41310, At1g09170, and At5g27000) are almost exclusively expressed in mature pollen (eFP browser, University of Toronto, Canada; Genevestigator, Nebion, Zurich, Switzerland). Combined with the observation that the sperm cells inside the pollen tube are encaged into a spindle-shaped microtubule structure (Laitiainen et al., 2002; Poulter et al., 2008) this led us to the hypothesis that KCH motors are the main actuator for the movement of sperm cells in the pollen tube.

In this study, we characterized the saltatory sperm movement in growing *Arabidopsis thaliana* pollen tubes, and propose a model to explain this motion by the function of KCH motors.

## Materials and Methods

### Plant Lines and Growth Conditions

For the pollen germination assays, pollen from plants of an histone 2B marker line (H2B-mTurquoise) kindly provided by Prof. Dr. Arp Schnittger (University Hamburg, Germany) and a tubulin A marker (TuA-mCherry) line kindly provided by Dr. Rene Schneider (MPIMP Golm, Germany) were used. All plants were sown on soil at 24°C. Illuminance was 100 μE s^−1^ m^−2^ for 18 h per day.

### Pollen Germination Assay

For the pollen germination assays, pollen from open but not yet withered *Arabidopsis thaliana* flowers were used. The anther was prepared and the pollen was placed on a gel cushion of pollen germination medium (0.01% H_3_BO_3_, 4 mM CaCl_2_, 1 mM KCl, 1 mM MgCl_2_, 18% sucrose, 5 mM MOPS pH7.0 with Tris, 0.5% agarose), which was placed on a gas-permeable adhesive foil (13-40-0516-24, Biolabproducts, Bebensee, Germany). The pollen was then trapped between the gel cushion and the slide. After an incubation period of 2 h at room temperature in darkness, pollen germination and sperm movement were imaged.

### Actin Transport Assay

The His-tagged protein OsKCH1 was expressed in the *Escherichia coli* strain BL21 (DE3) pRARE grown in LB medium and subsequently purified by affinity chromatography as previously described (Walter et al., 2015). Microtubules were polymerized as described before (Walter et al., 2012a,b). Actin filaments were polymerized from 3 μM chicken breast muscle actin in 10 mM 4-(2-hydroxyethyl)-1-piperazineethanesulfonic acid/NaOH pH 7.0, 100 mM KCl, 2 mM ATP, and 5 μM rhodamine phalloidin (R415, Invitrogen, Carlsbad, CA, USA) for 60 min at 4°C. Actin-Transport-Assays were performed as described in Walter et al. (2015).

### Imaging

Images were acquired by the NIS software packages (Nikon, Tokio, Japan) using an sCMOS camera (Andor Zyla 4.2, Oxford Instruments, Abingdon, UK) mounted on an inverted fluorescence microscope equipped with an autofocus system (Eclipse Ti, Nikon, Tokio, Japan). Cells were visualized using bright field or fluorescence epi-illumination with the respective filter sets.

### Evaluation of Run Velocity and Run Length

Positions of sperm cells and actin filaments were obtained using FIESTA tracking software as described before (Ruhnow et al., 2011). For each pollen, we calculated the displacement-weighted occurrence of sperm cells' velocities (Alper et al., 2013). Afterwards, we fitted a Gaussian fit function over the velocity data. From this fit, mean and standard deviation were extracted.

For each pollen, we determined the position of a sperm cell at defined time points. From this data, we calculated the so-called point-to-point displacements, i.e., the distance of the sperm cell at each point in time compared to the previous point in time. All positive displacements are forward movements, whereas negative displacements are backward movements. More than one displacement in either direction above a velocity threshold of 1 nm s^−1^ is taken together into a run (positive or negative). Displacements with a velocity smaller than 1 nm s^−1^ are seen as pauses. From displacement and time data, we calculated the velocities for each datapoint.

### Modeling

The model described in this paper is mainly based on the model described in Müller et al. (2008). Specific portions are adapted to fit the nature of the KCH motor proteins. We implemented the model with the following parameters: Each motor binds to a microtubule of the cargo with the binding rate π_0_ and detaches with the detachment rate ε_0_. Binding rates are increasing exponentially with increasing external force given by the detachment force F_d_. When bound to the microtubule, the motor moves toward the minus end of the respective microtubule with the forward velocity v_F_. This velocity decreases with external force and reaches zero at the stall force F_s_. Under superstall external forces, the motor moves toward the plus end of the respective microtubule with a slow velocity v_B_.

The motors bind to and detach from the cargo in a stochastic fashion, so the number of bound motors to each population of microtubules fluctuate with time.

$$\begin{array}{l} R_{tot}=n_{+}\epsilon +\left(N_{+}-n_{+}\right)\pi_{0}+n_{-}\epsilon \left(F\right)+\left(N_{-}-n_{-}\right)\pi_{0} \end{array}$$

For the modeling, we used the following parameters:

◦ Forward direction:
  – Stall force = 3 pN
  – Detachment force = 10 pN
  – Binding rate = 0.7 s^−1^
  – Unbinding rate = 1.7 s^−1^
  – Forward velocity = 0.08 μm s^−1^
  – Backward velocity = 0.008 μm s^−1^
◦ Backward direction:
  – Stall force = 3 pN
  – Detachment force = 10 pN
  – Binding rate = 0.6 s^−1^
  – Unbinding rate = 1.6 s^−1^
  – Forward velocity = 0.01 μm s^−1^
  – Backward velocity = 0.001 μm s^−1^
◦ Number of motors = 70

The Gillespie algorithm (Gillespie, 1976) is used to model the dynamic behavior of the motor protein. Therefore, the total rate is split into four parts: two for each motor direction as well as the cases binding and unbinding. Each of these events can happen with a different possibility but for each time interval, one of these events must happen.

The rate for one of these four events is based on its individual rate multiplied by the number of motors in this state. The binding rate is independent of the force acting on the motor, for both directions, respectively:

$$\begin{array}{l} \pi \left(F\right)=\pi_{0} \end{array}$$

However, the unbinding rate depends on the force acting on the motor, for both directions, respectively:

$$\begin{array}{l} \epsilon \left(F\right)=\epsilon_{0}\text{exp}\left(|F|/F_{d}\right) \end{array}$$

In contrast to Müller et al. (2008), there is no independent total number of plus and minus end-directed motors but just one number of total motors that can perform forward and backward runs. Thus, it exists only one type of unbound motors. This makes it still possible to apply the basic principles of the model. The velocity depends, similar to the unbinding rate, on the force

$$\nu \left(F\right)=\{\begin{array}{c} \nu_{F}\left(1-F/F_{s}\right)\text{ for }F\leq F_{s}\\ \nu_{B}\left(1-F/F_{s}\right)\text{ for }F\geq F_{s} \end{array}$$

From the relation of the cargo force:

$$\begin{array}{l} F_{c}=n_{+}F_{+}=n_{-}F_{-} \end{array}$$

and the cargo velocity:

$$\begin{array}{l} \nu_{c}\left(n_{+},n_{-}\right)=\nu_{+}\left(F_{c}/n_{+}\right)=\nu_{-}\left(-F_{c}/n_{-}\right) \end{array}$$

It is possible to derive expressions for the cargo force and the cargo velocity with two cases. For a stronger plus direction with *n*_+_*F*_*s*+_ > *n*_−_*F*_*s* −_

$$\begin{array}{l} F_{c}\left(n_{+},n_{-}\right)=\lambda n_{+}F_{s+}+\left(1-\lambda \right)n_{-}F_{s-}\\ \nu_{c}\left(n_{+},n_{-}\right)=\frac{\left(n_{+}F_{s+}-n_{-}F_{s-}\right)}{\frac{n_{+}F_{s}}{\nu_{F+}}+\frac{n_{-}F_{s}}{\nu_{B-}}}\\ \;\lambda =\frac{1}{1+\frac{n_{+}F_{s+}\nu_{B-}}{n_{-}F_{s-}\nu_{F+}}} \end{array}$$

For a stronger minus direction with *n*_−_*F*_*s*−_ > *n*_+_*F*_*s* +_

$$\begin{array}{l} F_{c}\left(n_{+},n_{-}\right)=\lambda n_{+}F_{s+}+\left(1-\lambda \right)n_{-}F_{s-}\\ \nu_{c}\left(n_{+},n_{-}\right)=\frac{\left(n_{+}F_{s+}-n_{-}F_{s-}\right)}{\frac{n_{+}F_{s}}{\nu_{B+}}+\frac{n_{-}F_{s}}{\nu_{F-}}}\\ \;\lambda =\frac{1}{1+\frac{n_{+}F_{s+}\nu_{F-}}{n_{-}F_{s-}\nu_{B+}}} \end{array}$$

Note that in the formulas for the second case, the expression for the cargo force is identical as before. However, in the other equations the forward velocity *v*_*F*+_ has to be replaced by its backward velocity *v*_*B*+_ and *v*_*B*−_ by *v*_*F* −_.

Due to the use of the Gillespie algorithm, the time interval of the simulation is not uniform. It is the key idea of the Gillespie algorithm that the time interval is based on an exponentially distributed random function. To obtain comparable datasets, the simulated datasets were downsampled to the frame-rate of the experimental data. When necessary, a linear interpolation was used to determine the position of the simulated sperm cells at a given time point, taking the neighboring simulation points into account.

## Results

### Sperm Cells Are Transported With a Higher Velocity and With Longer Runs Toward the Tube Apex Than Toward the Pollen Grain

**Figure 1:**
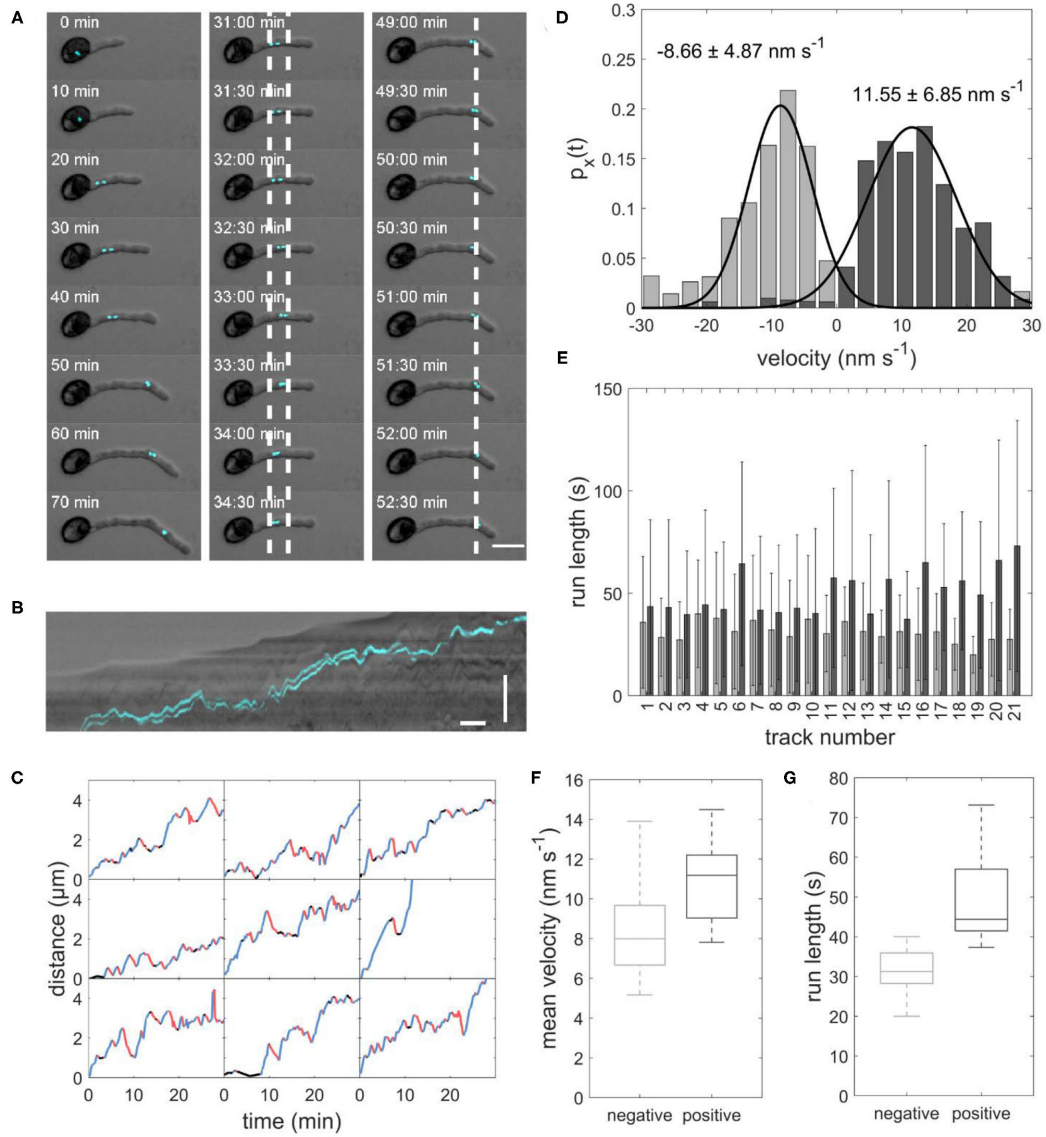
**(A)** Image sequence of sperm cells (cyan) transported inside a growing pollen tube. Left panel: The sperm cells move from the pollen grain to the tip of the growing pollen tube. Central panel: Example of sperm cells moving forward and backward between the two dotted lines. Right panel: Example of sperm cells staying stationary for several minutes at the dotted line. Scale bar: 25 μm. **(B)** Kymograph of sperm cells transported inside a pollen tube. Horizontal scale bar: 5 min, vertical scale bar: 30 μm. **(C)** Distance-over-time plots for nine sperm cells. Blue: forward runs, red: backward runs, black: pauses. **(D)** Displacement-weighted velocity distributions for forward (dark gray) and backward (light gray) runs of one sperm cell. **(E)** Barplot of the run lengths for each sperm cell in forward (dark gray) and backward (light gray) directions. **(F)** Boxplot of mean forward (dark gray) and backward (light gray) velocities calculated from the gaussian fits over displacement-weighted velocity distributions (*n* = 21, *p* < 0.05). **(G)** Boxplot of the mean run lengths in forward (dark gray) and backward (light gray) directions (*n* = 21, *p* < 0.05).

In flowering plants, sperm cells are transported inside the growing pollen tube from the pollen grain toward the tube apex. To investigate the exact path of the moving sperm cells, time-lapse microscopy of germination assays was performed with pollen from an mTurquoise-histone 2B *Arabidopsis thaliana* marker line. Since this histone is highly expressed in sperm cells, the generative cells could be visualized and tracked during the experiment (Figures 1A,B and Supplementary Movie 1).

As expected, we found a net movement of the sperm cells from the pollen grain toward the tube apex in all observed pollen. We determined an average sperm cell velocity of 7.0 ± 4.2 μm h^−1^ (*n* = 21). Remarkably, the sperm cells in all observed pollen did not continuously move toward the tube apex but performed several forward movements toward the apex and backward movements toward the pollen grain with intermediate pauses (Figures 1A–C). Furthermore, the sperm cells are not permanently arranged one behind the other, but sometimes change their position (Supplementary Movie 2). This is of particular interest as this changes the orientation of the cage at the same time apparently without affecting the transport pattern.

Duration and velocity of each run and were highly variable. We evaluated the velocity of each forward and backward run. Figure 1D shows an example histogram with mean velocity and standard deviation for all forward runs (11.55 ± 6.85 nm s^−1^) and all backward runs (−8.66 ± 4.87 nm s^−1^) the sperm cells performed in one pollen tube. In all evaluated pollen, the mean velocity of the forward runs was higher than the mean velocity of the backward runs (Table 1). The mean velocity of sperm cells in 21 evaluated pollen was −9.06 ± 4.45 nm s^−1^ for backward runs and 10.85 ± 1.93 nm s^−1^ for forward runs. The forward velocities were significantly higher than the backward velocities (*p* < 0.05; *n* = 21; Figure 1F). Moreover, we found that for each pollen, the runs in the forward direction (44.89 ± 39.69 s, *n* = 21) are longer than in the backward direction (32.03 ± 25.15 s, *n* = 21; Figures 1E,G).

**Table 1:** Mean velocity and standard deviation for all sperm cell tracks in forward and backward direction.

**Track number**	**Mean forward velocity ± SD (nm s^−1^)**	**mean backward velocity ± SD (nm s^−1^)**
1	8.74 ± 4.96	−6.69 ± 3.66
2	7.81 ± 4.25	−7.41 ± 5.27
3	9.07 ± 5.44	−6.60 ± 2.56
4	9.31 ± 4.91	−7.66 ± 3.72
5	11.86 ± 6.47	−11.78 ± 7.17
6	14.49 ± 9.02	−26.17 ± 23.79
7	12.08 ± 7.75	−9.90 ± 5.70
8	13.82 ± 8.70	−8.99 ± 4.49
9	11.55 ± 6.85	−8.66 ± 4.87
10	8.53 ± 4.88	−7.99 ± 4.41
11	11.25 ± 5.57	−13.90 ± 11.82
12	12.57 ± 6.01	−9.60 ± 4.65
13	8.89 ± 5.46	−6.35 ± 3.31
14	10.86 ± 6.76	−5.79 ± 2.92
15	9.42 ± 4.93	−8.17 ± 4.58
16	11.18 ± 6.06	−9.19 ± 5.14
17	13.30 ± 7.28	−5.46 ± 2.31
18	8.93 ± 4.79	−6.92 ± 2.55
19	9.74 ± 5.15	−5.17 ± 1.63
20	12.84 ± 7.10	−7.74 ± 4.60
21	11.66 ± 6.47	−10.16 ± 6.42

**Figure 2:**
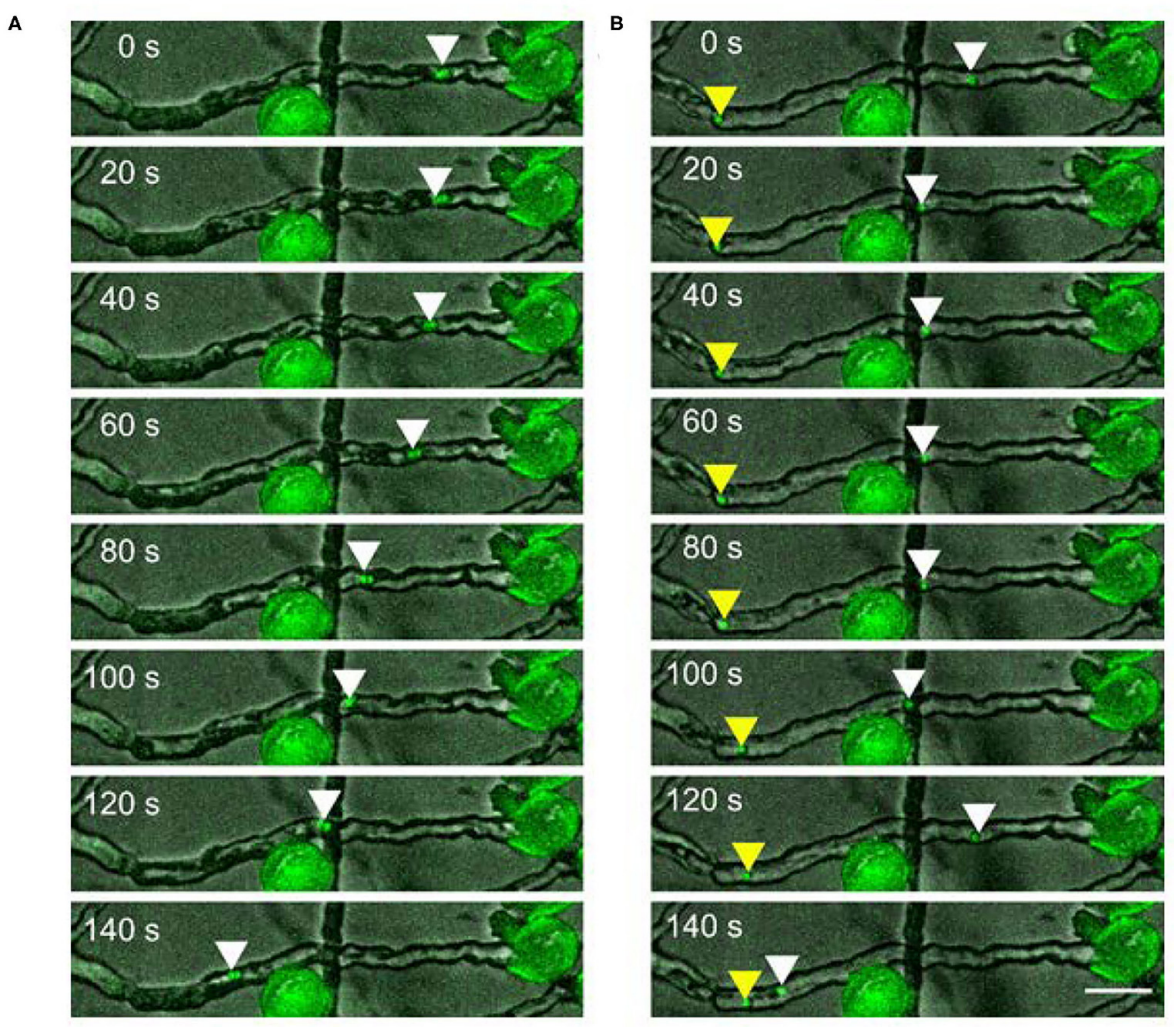
Two sperm cells moving **(A)** together or **(B)** independently upon dissociation in a pollen tube. Scale bar: 30 μm. Arrowheads mark the position of sperm cells.

We also occasionally observed the dissociation of the male germ unit. In this case, each sperm cell moved independently inside the pollen tube. The movement pattern of each sperm cell is comparable to the movement of the connected sperm cells. It shows forward and backwards runs with intermediate pauses (Figure 2 and Supplementary Movie 3).

### The Putative Function of KCH Motor Proteins in Sperm Cell Transport

**Figure 3:**
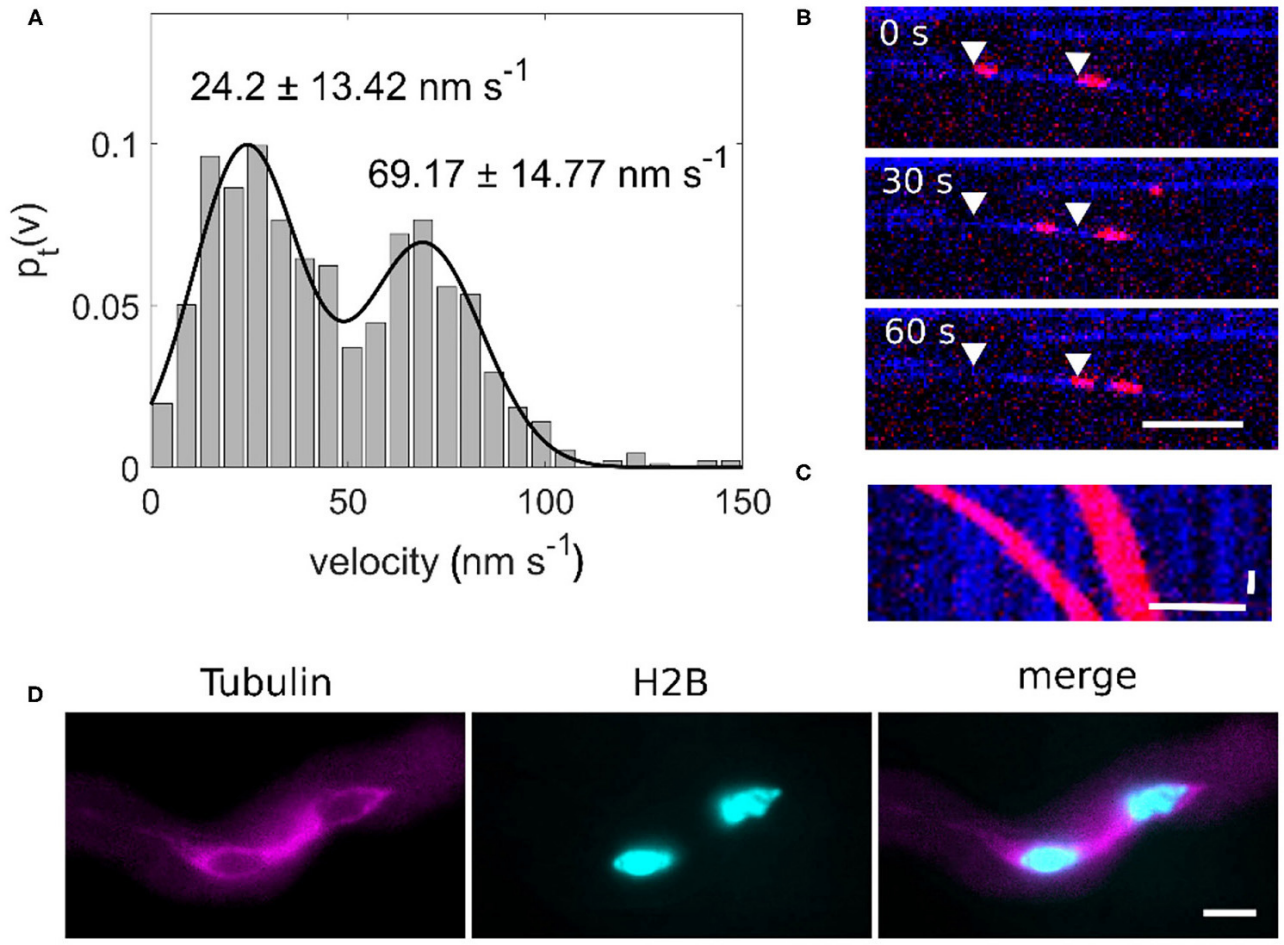
**(A)** Bimodal velocity distribution of point-to-point velocities of an actin transport assay (*n* = 58). **(B)** Image sequence of an OsKCH1 actin transport assay at different timepoints. Two actin filaments (red) are transported along one microtubule (blue) in two distinct velocities. Arrowheads mark the starting points of the actin filaments. Scale bar: 5 μm. **(C)** Kymograph of an actin transport assay. Horizontal scale bar 5 μm, vertical scale bar: 10 s. **(D)** Microtubule cage (magenta) surrounding the sperm cells (cyan) within a pollen tube. Scale bar: 10 μm.

We performed *in vitro* assays with OsKCH1 to characterize the movement parameters of OsKCH1 and compare them with those of sperm cell transport. The actin transport assays show that OsKCH1 transports actin filaments at two different velocities along microtubules as previously described (Walter et al., 2015). The slower population of actin filaments is transported with a velocity of 24.2 ± 13.42 nm s^−1^ and the faster population is transported with a velocity of 69.17 ± 14.77 nm s^−1^ (mean ± s.d., *N* = 58 total transport events, Figures 3A–C).

**Figure 4:**
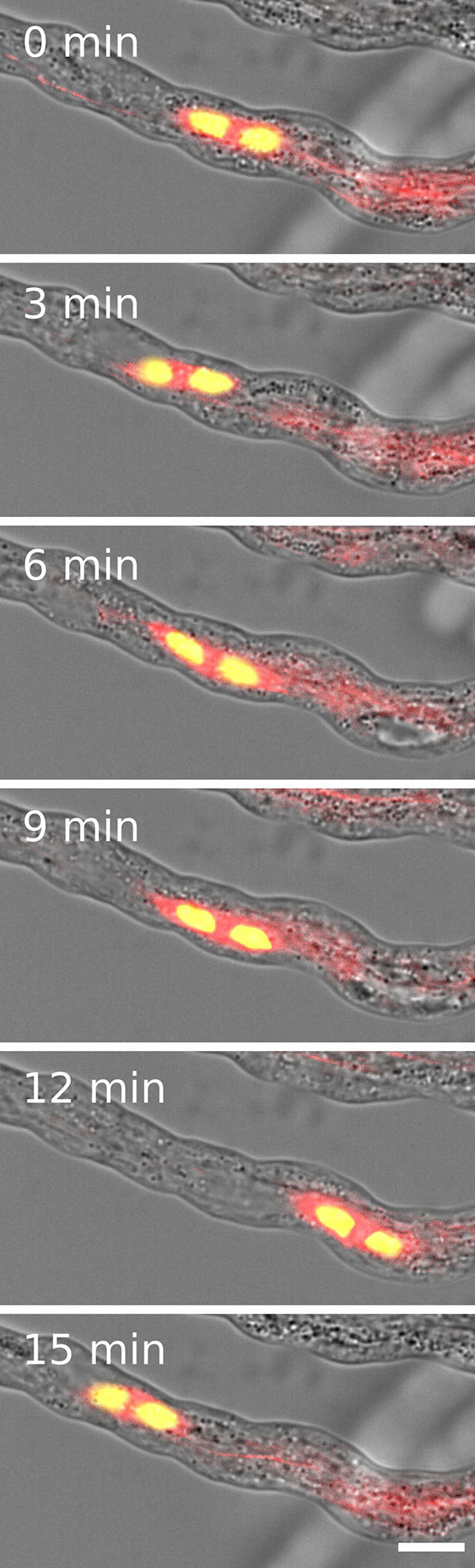
Two sperm cells (yellow) are transported inside a microtubule cage (red) within a growing pollen tube. Scale: 10 μm.

Since we also observed two velocities for the forward and backward movement of sperm cells and four KCHs are expressed only in mature pollen, we hypothesized that KCHs play a role in the transport of sperm cells inside the pollen tube. KCH kinesins are interconnecting microtubules and actin filaments. Actin filaments are usually bundled in long cables at the cell cortex and oriented in a polar way in pollen tubes where they play a major role in vesicle transport (Cai and Cresti, 2009). In contrast, the role of microtubules and kinesin-mediated transport in pollen tubes is not yet fully understood. Performing pollen germination assays with pollen from a Tubulin-mCherry *Arabidopsis thaliana* marker line showed a cage of microtubules around sperm cells (Figure 3D) which moves together with the sperm cells in a saltatory manner (Figure 4 and Supplementary Movie 4).

### Movement of Sperm Cells Inside Pollen Tubes Can Be Described With a Tug-of-War Model

Based on our observations of the saltatory sperm movement, the microtubule cage, and the characteristic features of KCH motors, we adapted a model by Müller et al. (2008) to describe KCH-driven sperm cell transport. The original model simulated the movement of small cargoes transported by groups of motor proteins that exert force in opposing directions resulting in subsequent forward and backward runs that resemble the saltatory movements of sperm cells.

**Figure 5:**
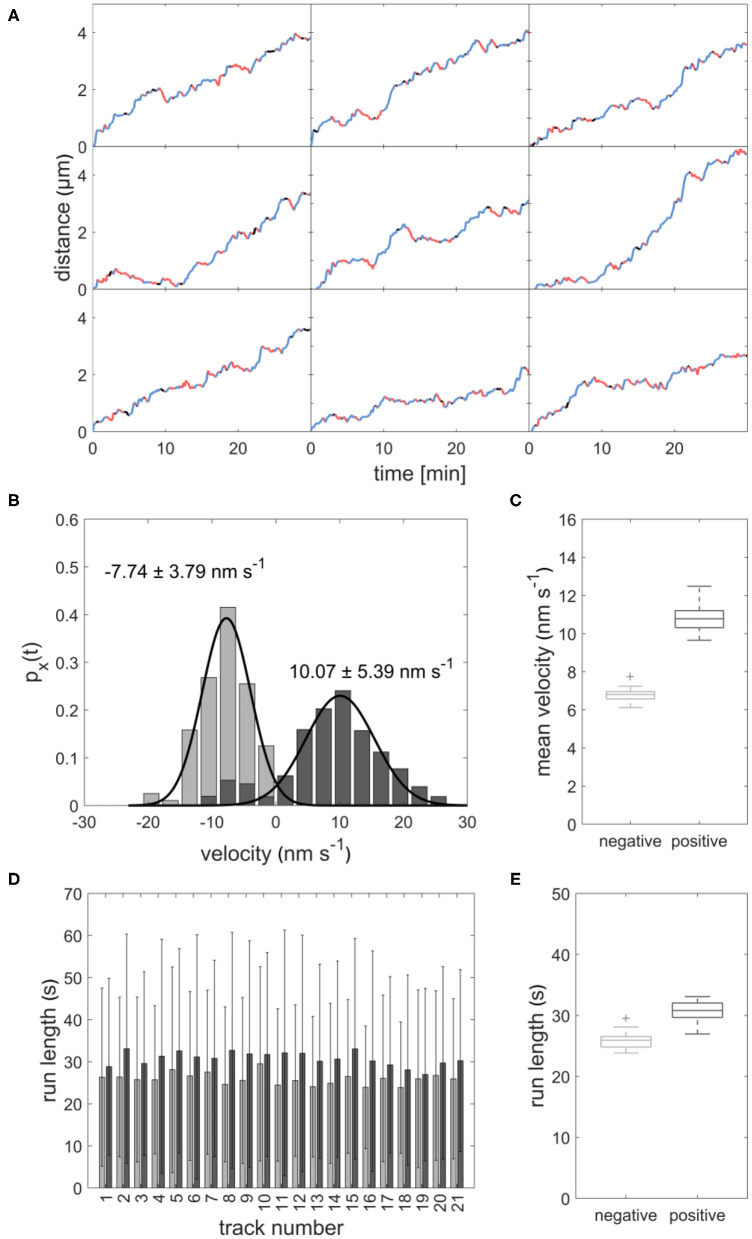
**(A)** Distance-over-time plots for 9 modeled sperm cells. Blue: forward runs, red: backward runs, black: pauses. **(B)** Displacement-weighted velocity distributions for forward (dark gray) and backward (light gray) runs of one modeled sperm cell. **(C)** Boxplot of mean forward (dark gray) and backward (light gray) velocities calculated from the gaussian fits over displacement-weighted velocity distributions (*n* = 21, *p* < 0.05). **(D)** Barplot of the run lengths for each modeled sperm cell in forward (dark gray) and backward (light gray) directions. **(E)** Boxplot of the mean run lengths of the modeled sperms in forward (dark gray) and backward (light gray) directions (*n* = 21, *p* < 0.05).

**Figure 6:**
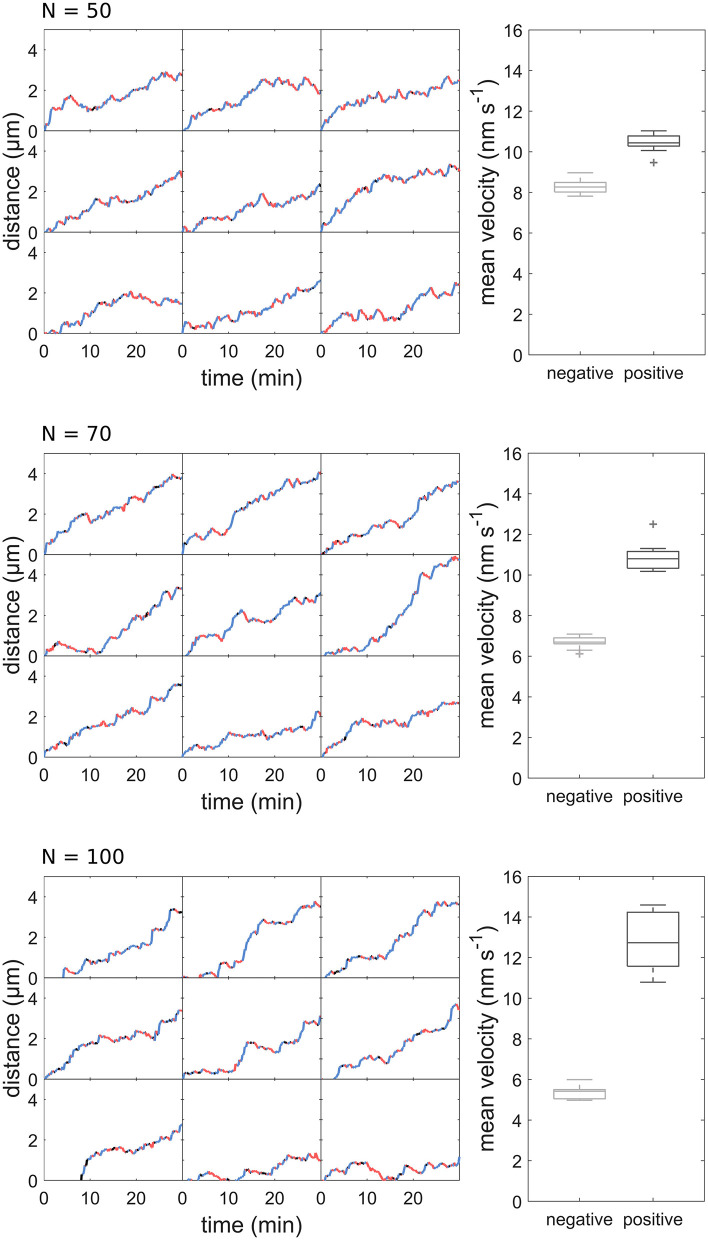
Distance-over-time plots and mean velocity of sperm cell movement patterns modeled with different motor numbers.

Using our model, we created 21 sample datasets, which were evaluated in the same way as the experimental data. The individual tracks of the model data are comparable to those of the experimental data (Figure 5A). As with the experimental data, the runs varied in length and velocity. The mean velocity was 10.83 ± 0.76 nm s^−1^ for forward runs and 6.77 ± 0.38 nm s^−1^ for backward runs (Figures 5B,C). The mean run length was 30.76 ± 1.66 s for forward runs and 25.91 ± 1.40 s for backward runs (Figures 5D,E). Both run length and velocity in the forward direction was significantly higher than in the backward direction (*p* < 0.05; *N* = 21). The simulated values correspond to the observed *in vivo* data (Figures 1D–G). To rule out an effect of the unknown motor number on the transport patterns, we performed the modeling with different numbers of motor proteins, which had no influence on the transport pattern (Figure 6).

## Discussion

### Proposed Model for Sperm Cell Transport in Pollen Tubes

We found that sperm cells are transported in *Arabidopsis thaliana* pollen tubes in a saltatory manner. Forward movements toward the pollen tube tip of the sperm cells are typically longer and faster than backward runs toward the pollen grain. This necessarily results in a net movement direction in the forward direction. By comparing experimental data with model data, we showed that this movement can be described by a tug-of-war mechanism of cortical KCHs acting on the microtubule cage surrounding the sperm cells.

**Figure 7:**
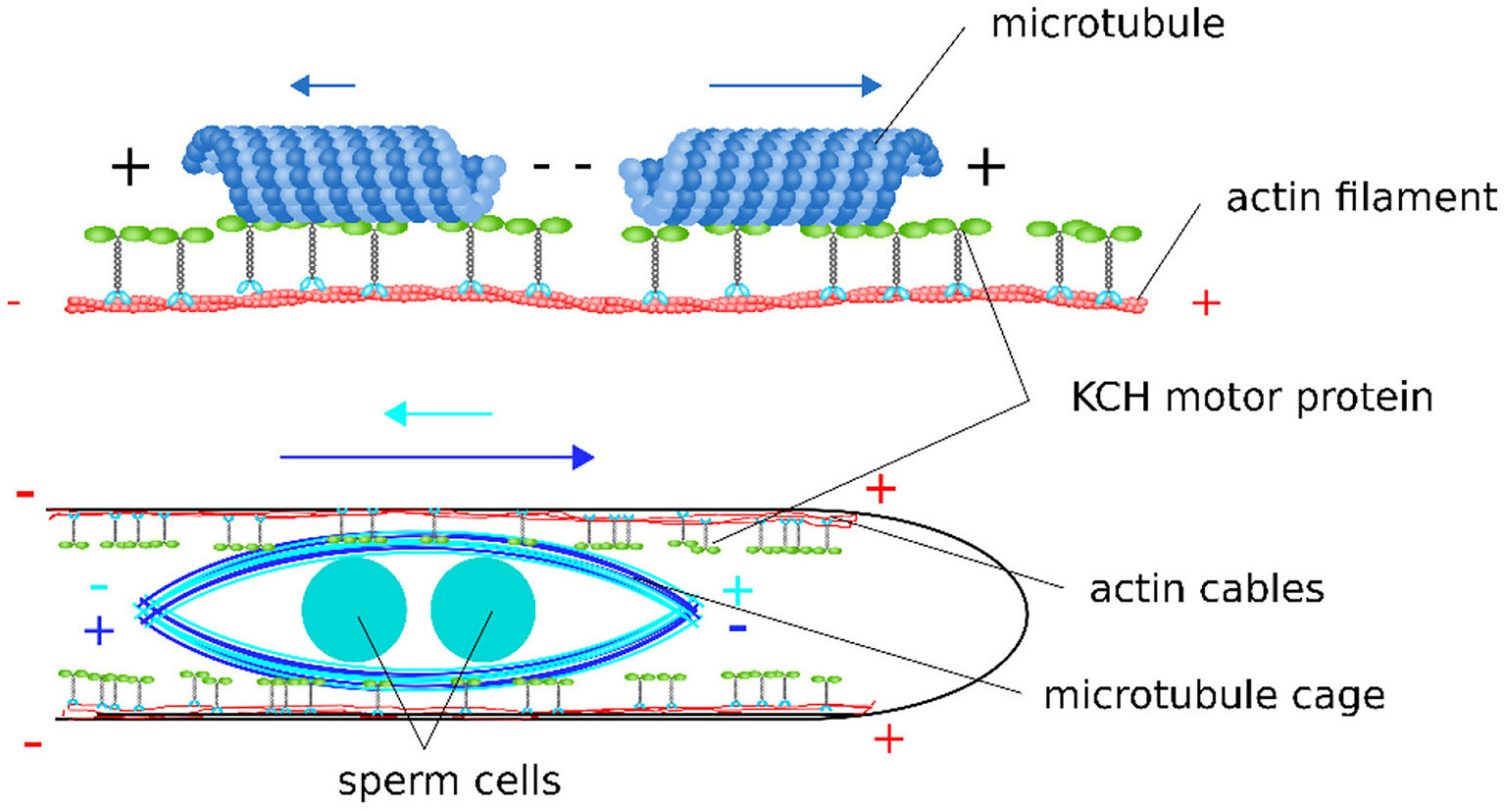
Scheme of the proposed model for sperm cell transport mediated by KCHs statically bound to cortical actin cables. Depending on their polar orientation, microtubules are transported in opposite directions and at different velocities along actin filaments. As the sperm cells are encaged into a microtubule structure, this bidirectionality leads to a tug-of-war scenario when sperm cells are transported along the polar, cortical actin cables within the pollen tube.

We propose the following model for the sperm cell movement in pollen tubes: sperm cells are enclosed in a cage formed by microtubules with a random polar orientation so that there are plus and minus ends at each end of the cage. Parallel actin cables with the plus end pointing toward the tube apex are located at the cortex of the pollen tube (Lenartowska and Michalska, 2008). The microtubules forming the sperm cell cage and the cortical actin cables are interconnected by KCH kinesins. The KCHs are statically bound to the actin cables with their calponin homology domain as previously shown (Walter et al., 2015). The kinesins are moving along the microtubules of the sperm cell cage with their motor domain, resulting in a movement of the whole cage (Figure 7). The mechanism resembles an inverse actin transport (Figure 7): Actin filaments are firmly bound and microtubules are transported.

We assume that the microtubules are randomly arranged around the sperm cells, i.e., there are microtubules with their minus end pointing toward the pollen tube tip (in the forward direction) and microtubules with their minus end pointing toward the pollen grain (in the backward direction). Since KCHs are minus-end directed kinesins, they shift the microtubules either in the forward direction or in the backward direction, depending on the respective orientation of the microtubule they bound to. Accordingly, the mobile cage would be move, because the kinesins are firmly anchored to the cortical actin filaments. If by chance all kinesins were to bind exclusively to the microtubules with their minus ends pointing toward the pollen grain, a situation would arise as shown on the right panel of Figure 7. The KCHs would all move toward the minus end, pushing the sperm cell cage and sperm cells in the forward direction. In *in vitro* experiments with OsKCH1, it was found that the transport velocity depends on the orientation of the microtubule in relation to the actin filament. Thus, for the case described above, we assume that this represents the “more favorable” orientation and that the sperm cell cage moves at a fast speed toward the pollen tube tip. For the opposite case, i.e., if by chance all kinesins bind to the microtubules whose minus end points toward the pollen tube tip, the sperm cell cage is pushed toward the pollen grain accordingly. For this case, the orientation of microtubules and actin filaments is as shown in Figure 7 for the left microtubule and the sperm cells move at slow speed in the backward direction. During the transport process, all combinations of bound kinesins to microtubules of one orientation as well as the other are possible and determines the speed and direction with which the sperm cells move. The forward movement is always faster than the backward movement. This results in a net movement toward the pollen tube tip.

The model is in agreement with the results of several studies on microtubules and kinesins in the pollen tube. A spindle-shaped microtubule structure, similar to the one we found in Arabidopsis, has also been found in other plants like tobacco (Åström et al., 1995), papaver (Poulter et al., 2008), and *Tradescantia virginiana* (Palevitz and Cresti, 1988). A function has not yet been assigned to these microtubules. However, it has been confirmed several times that a disruption of the microtubules in the pollen tube contributes to the retarding of sperm cell transport. This is true both for disruption with the help of substances such as oryzalin (Åström et al., 1995) or colchicine (Heslop-Harrison et al., 1988) as well as for cold treatment (Åström et al., 1991). Kinesins have already been detected in the pollen tube by immunostaining (Cai et al., 1993), although only in the tube apex. Nevertheless, Romagnoli et al. (2003) have shown in *in vitro* studies that organelles from the pollen tube are transported via kinesins and propose that kinesins play a role in organelle transport in addition to myosins. The overall sparse presence of microtubules in the pollen tube emphasizes the unique importance of the described microtubule cage. It is fair to speculate, that microtubule-associated processes have been gradiently reduced in pollen tubes in order to avoid effects on the transport of the sperm cells as described in our model.

In *in vitro* studies with OsKCH1, a KCH from rice, it has been shown that OsKCH1 is a non-processive motor protein (Walter et al., 2015), i.e., it does not move along microtubules over a longer period of time, but detaches from the microtubule after each power stroke and has to bind again for another power stroke. For the model, we have assumed that the kinesins transporting the sperm cells are also non-processive. OsKCH2, however, is processive but also transports actin filaments at two different velocities comparable to OsKCH1 in an actin transport assay (Walter et al., 2015; Tseng et al., 2018). It can, therefore, be assumed that the movement of the sperm cells can also be reproduced under the assumption of a processive motor.

According to expression studies, KCH1, 2, 5, and 6 in *Arabidopsis* are almost exclusively expressed in the pollen tube. Therefore, we consider them as promising candidates for the transport of sperm cells in the pollen tube. Localization studies and pollen germination assays with corresponding KCH knockout mutant lines are important next steps toward revealing the exact transport mechanism of sperm cells in pollen tubes.

Our model is in contrast to the common hypothesis that sperm cells are transported as passive cargo attached to the actively transported vegetative nucleus. This hypothesis was mainly based on the identification of the cytoplasmic projection connecting the sperm cells with the vegetative nucleus. It was shown that this cytoplasmic projection plays some role in the sperm cell guidance and might also contribute to some other regulatory mechanisms inside the pollen (McCue et al., 2011). Nevertheless, we were able to show that upon dissociation of the male germ unit the sperm cells are still moving inside the pollen tube which would be impossible if the sperm cells were merely dragged along as a passive cargo. In addition, we have observed cases in which the sperm cells change their position and are still transported afterwards, which also speaks against the theory of sperm cells as passive cargo. Our model explains how the sperm cells can be actively transported by KCHs anchored on cortical actin cables acting on the spindle-shaped microtubule cage.

## Data Availability Statement

The raw data supporting the conclusions of this article will be made available by the authors, without undue reservation.

## Author Contributions

WW and SS designed the experiments and wrote the manuscript. SS, FM, and EH carried out the experiments. SS and JS generated our model and analyzed the data. All authors discussed the results. All authors have read and agreed to the published version of the manuscript.

## Conflict of Interest

The authors declare that the research was conducted in the absence of any commercial or financial relationships that could be construed as a potential conflict of interest.

## Supplementary Material

Supplementary Movie 1 Saltatory movement of sperm cells (cyan) transported inside a growing pollen tube.

Supplementary Movie 2 Two sperm cells change their position during transport inside a growing pollen tube.

Supplementary Movie 3 Two sperm cells move independently upon dissociation in a pollen tube.

Supplementary Movie 4 Two sperm cells (yellow) are transported inside a microtubule cage (red) within a growing pollen tube.
